# Supplementary figures and images for: Ferroptotic cardiomyocyte-derived exosomes promote cardiac macrophage M1 polarization during myocardial infarction
Source: PeerJ. 2022 Jul 6;10:e13717. doi: 10.7717/peerj.13717 (PMC9270880; doi:10.7717/peerj.13717)

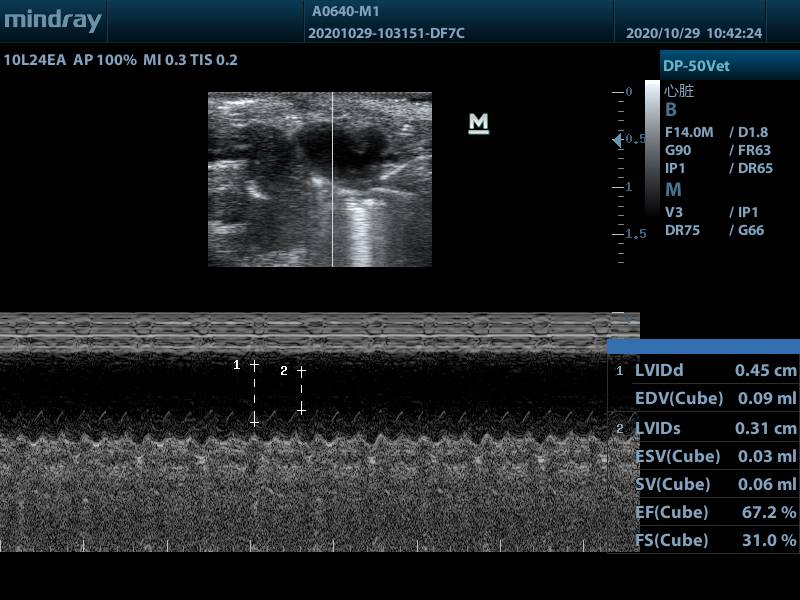

Supplement: Supplemental Information 2 [file peerj-10-13717-s002.zip › 20220316 Raw data/Fig 1A-1B Echocardiographic examination/MI 1.JPG]

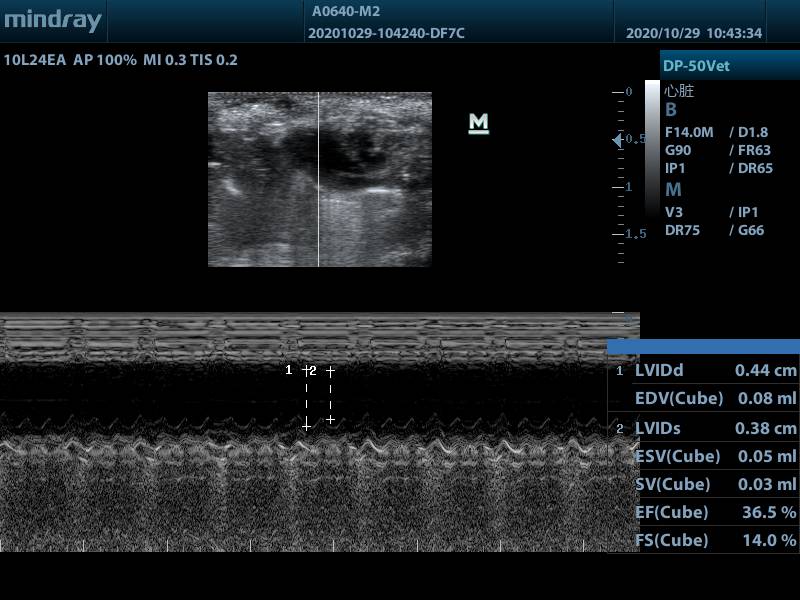

Supplement: Supplemental Information 2 [file peerj-10-13717-s002.zip › 20220316 Raw data/Fig 1A-1B Echocardiographic examination/MI 2.JPG]

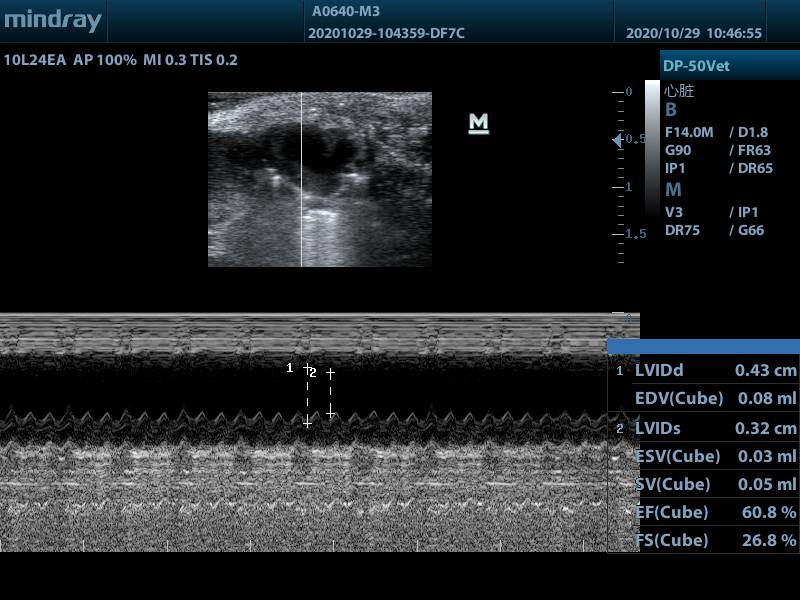

Supplement: Supplemental Information 2 [file peerj-10-13717-s002.zip › 20220316 Raw data/Fig 1A-1B Echocardiographic examination/MI 3.JPG]

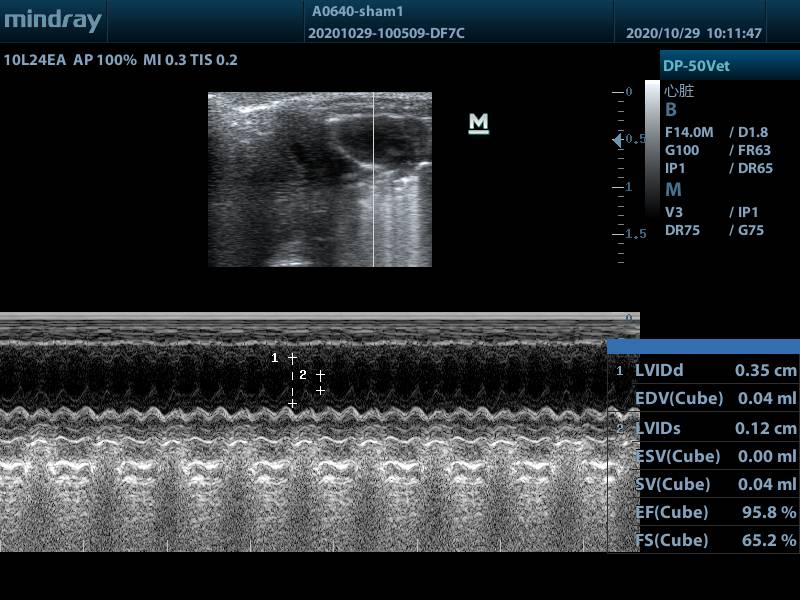

Supplement: Supplemental Information 2 [file peerj-10-13717-s002.zip › 20220316 Raw data/Fig 1A-1B Echocardiographic examination/sham 1.JPG]

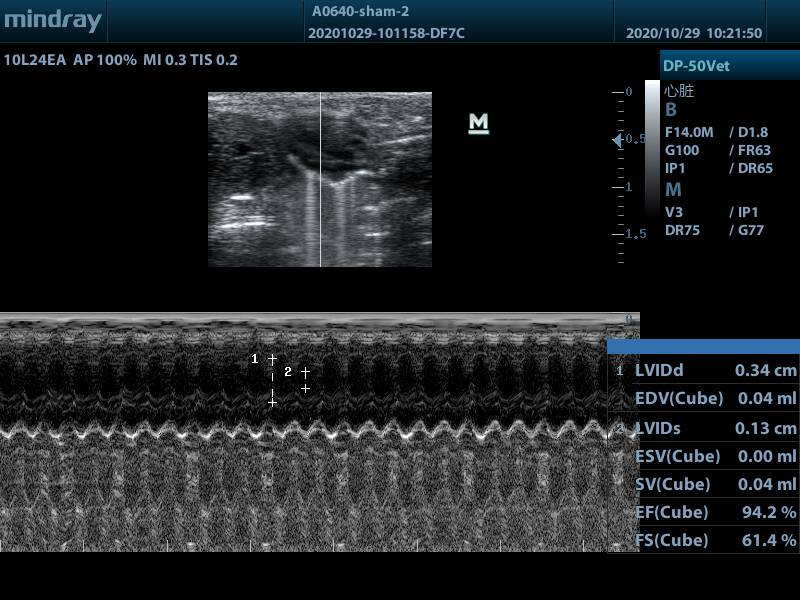

Supplement: Supplemental Information 2 [file peerj-10-13717-s002.zip › 20220316 Raw data/Fig 1A-1B Echocardiographic examination/sham 2.JPG]

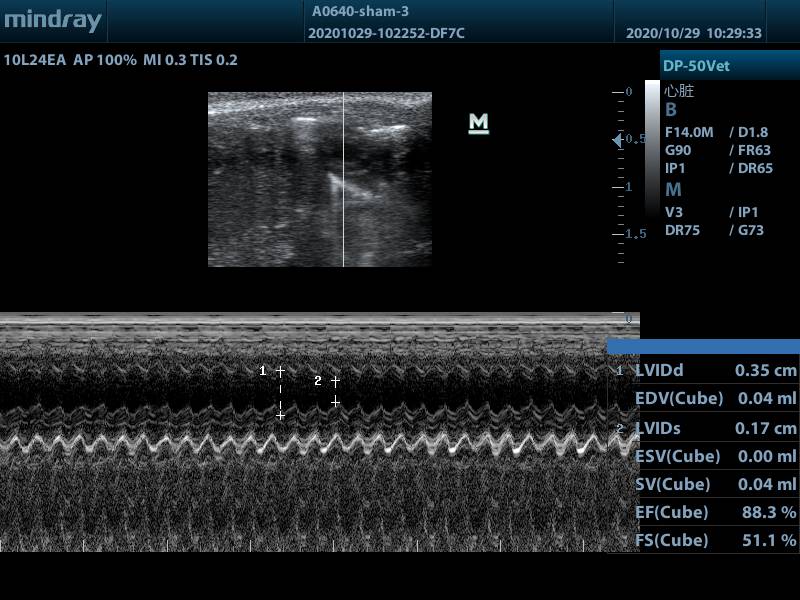

Supplement: Supplemental Information 2 [file peerj-10-13717-s002.zip › 20220316 Raw data/Fig 1A-1B Echocardiographic examination/sham 3.JPG]

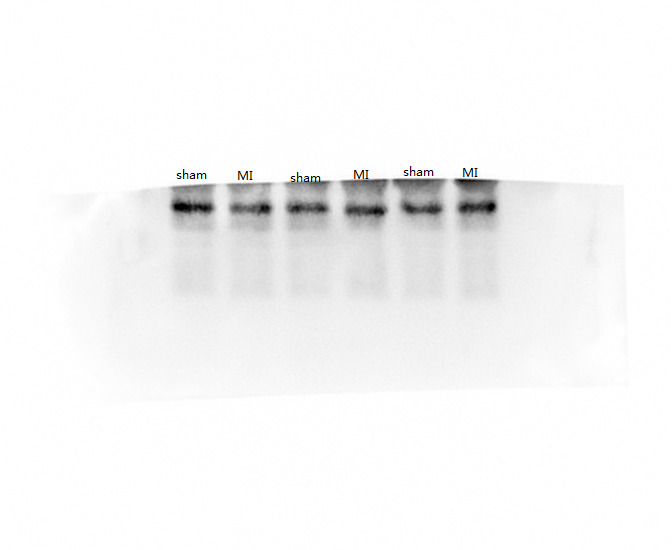

Supplement: Supplemental Information 2 [file peerj-10-13717-s002.zip › 20220316 Raw data/Fig 1F WB gel/GAPDH-IL-10.tif]

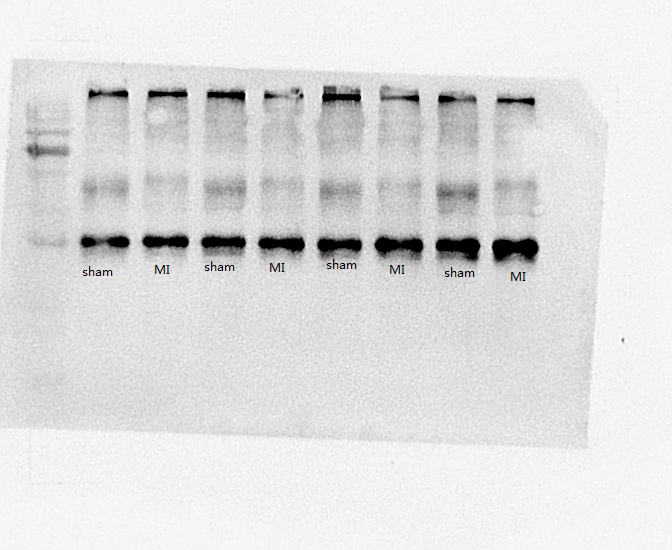

Supplement: Supplemental Information 2 [file peerj-10-13717-s002.zip › 20220316 Raw data/Fig 1F WB gel/GAPDH-NOS2.tif]

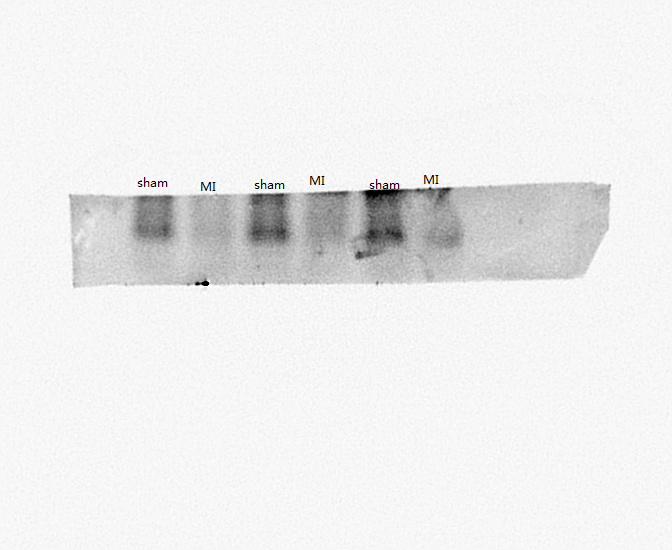

Supplement: Supplemental Information 2 [file peerj-10-13717-s002.zip › 20220316 Raw data/Fig 1F WB gel/IL-10.tif]

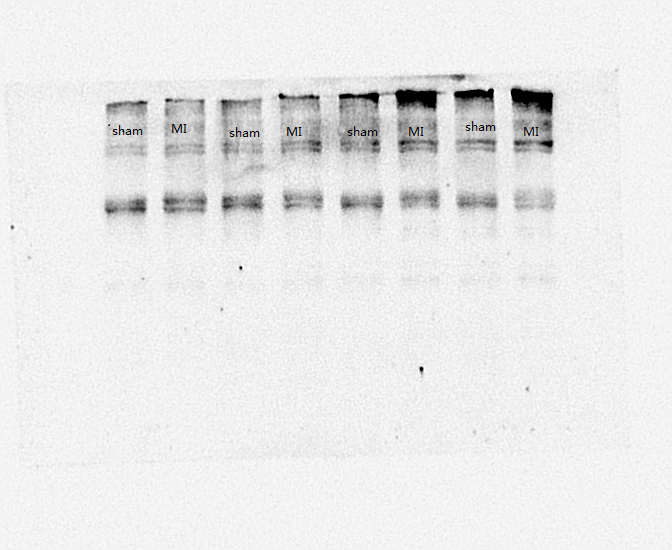

Supplement: Supplemental Information 2 [file peerj-10-13717-s002.zip › 20220316 Raw data/Fig 1F WB gel/NOS2.tif]

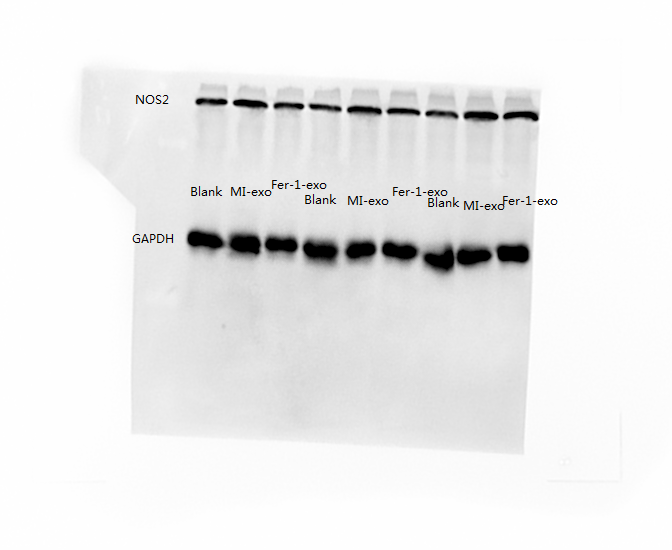

Supplement: Supplemental Information 2 [file peerj-10-13717-s002.zip › 20220316 Raw data/Fig 3B WB gel/GAPDH and NOS2.tif]

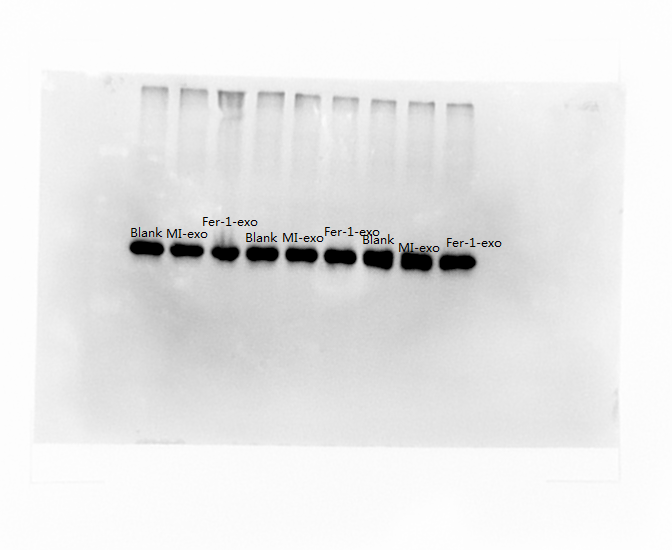

Supplement: Supplemental Information 2 [file peerj-10-13717-s002.zip › 20220316 Raw data/Fig 3B WB gel/GAPDH-IL-10.tif]

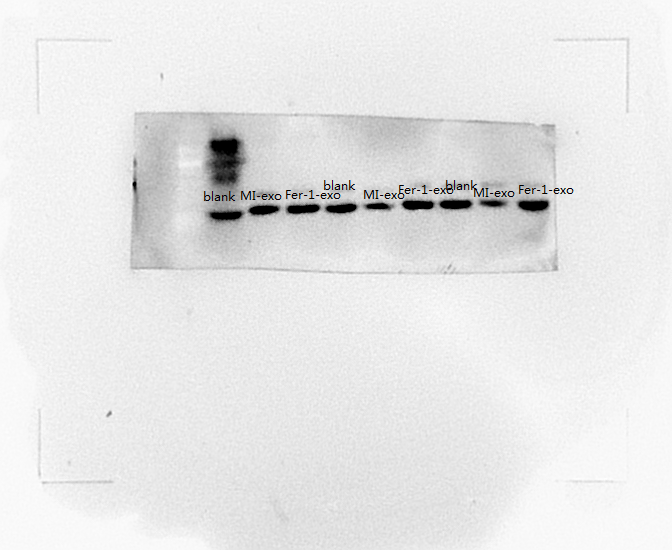

Supplement: Supplemental Information 2 [file peerj-10-13717-s002.zip › 20220316 Raw data/Fig 3B WB gel/IL-10.tif]

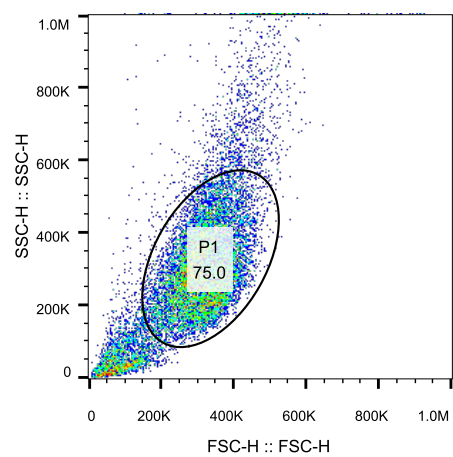

空白组-1

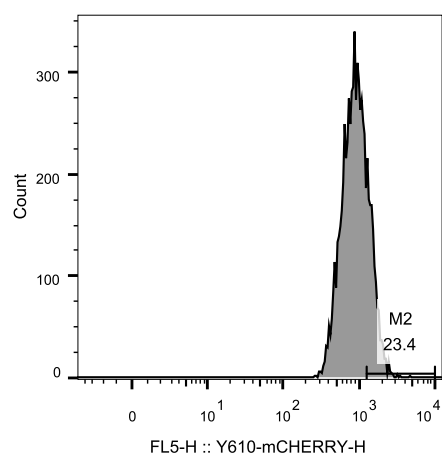

M2

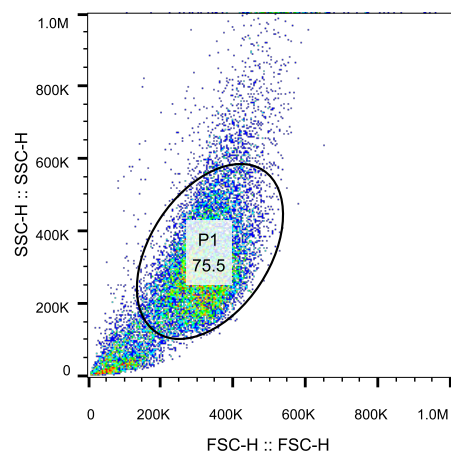

空白组-2

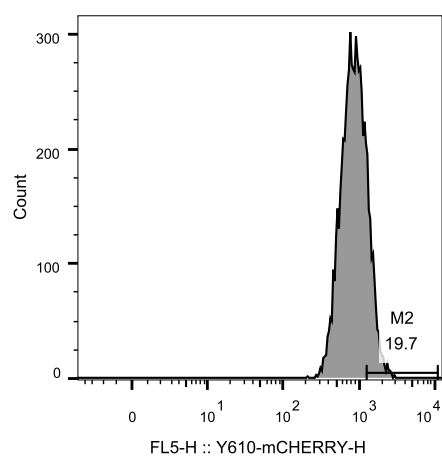

M2

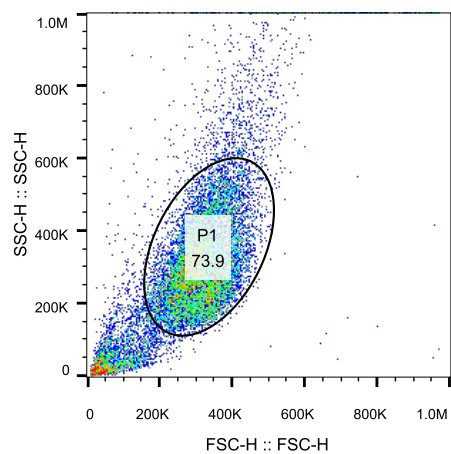

空白组-3

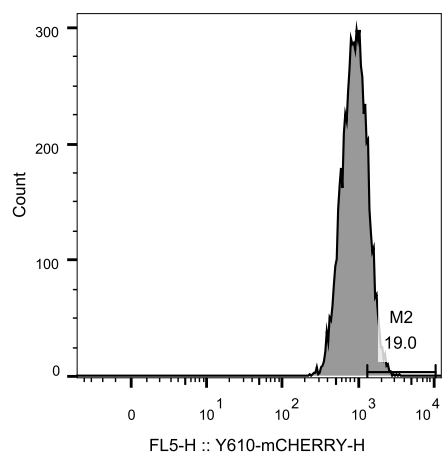

M2

Supplement: Supplemental Information 2 [file peerj-10-13717-s002.zip › 20220316 Raw data/Fig 3C FCM/Blank.pdf]

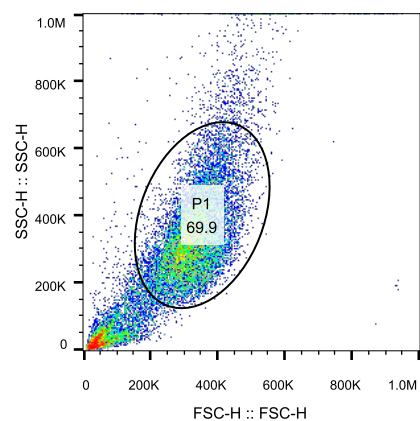

Fe组-1

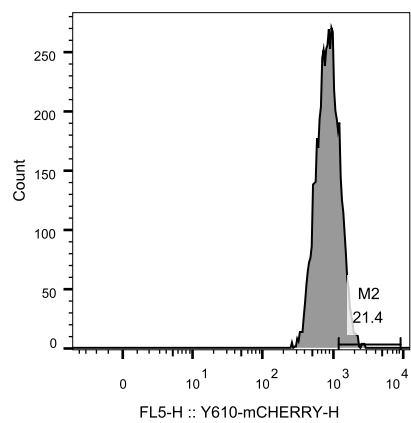

M2

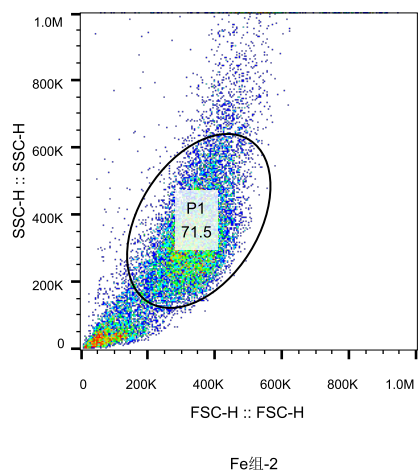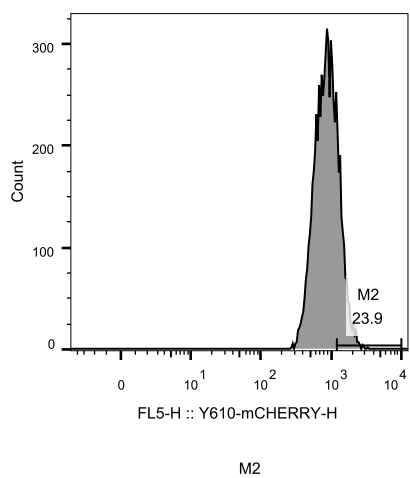

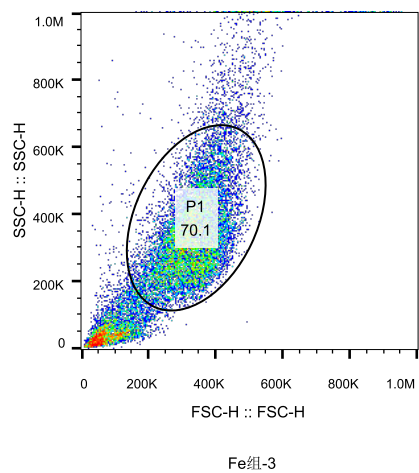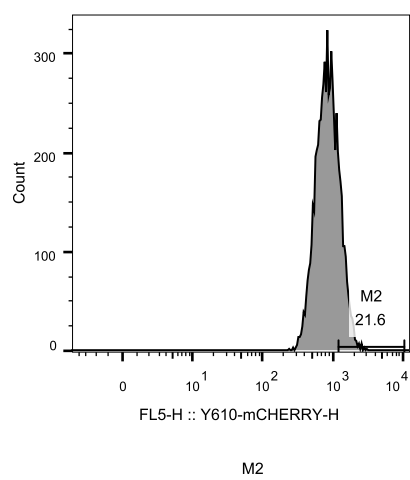

Supplement: Supplemental Information 2 [file peerj-10-13717-s002.zip › 20220316 Raw data/Fig 3C FCM/Fer-1-exo.pdf]

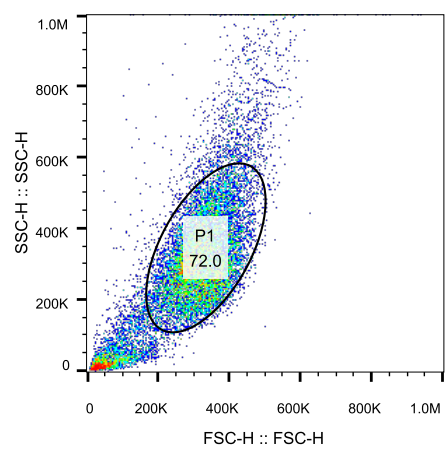

对照组-1

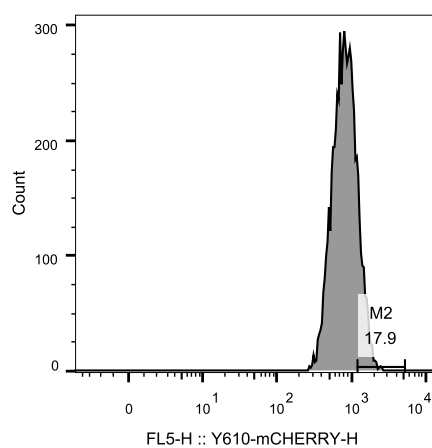

M2

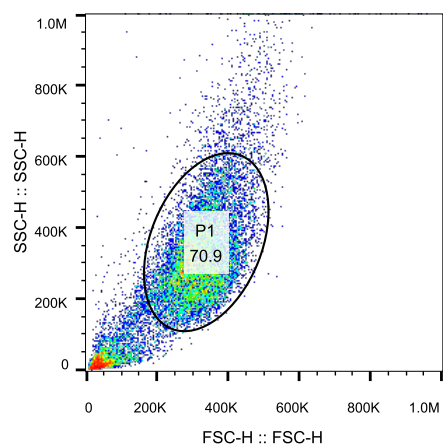

对照组-2

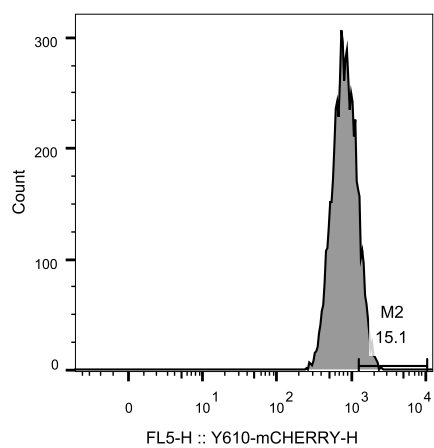

M2

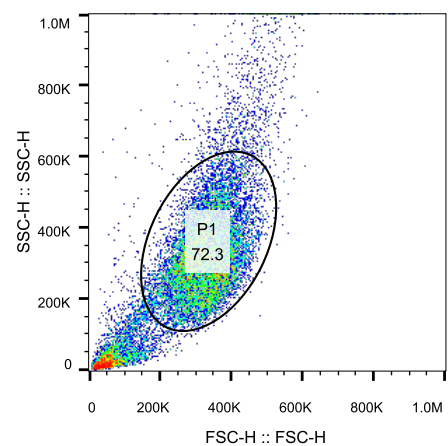

对照组-3

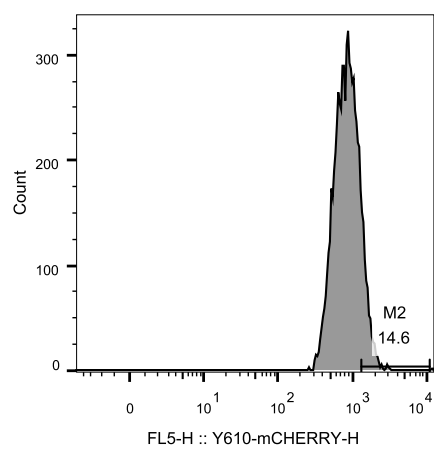

M2

Supplement: Supplemental Information 2 [file peerj-10-13717-s002.zip › 20220316 Raw data/Fig 3C FCM/MI-exo.pdf]

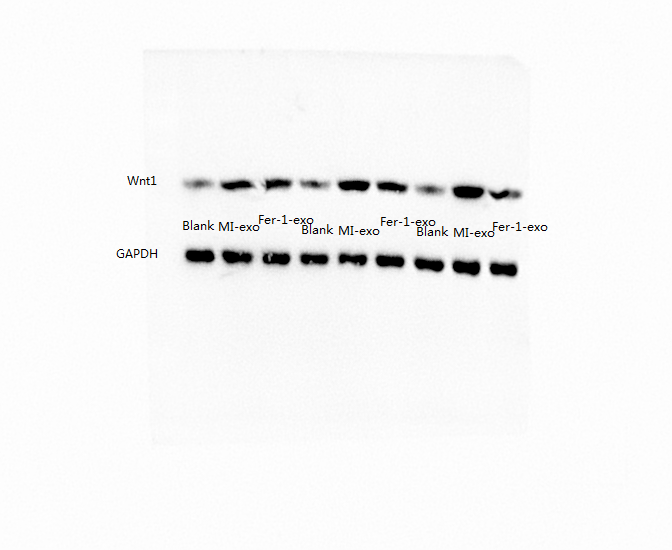

Supplement: Supplemental Information 2 [file peerj-10-13717-s002.zip › 20220316 Raw data/Fig 4D WB gel/GAPDH and Wnt1.tif]

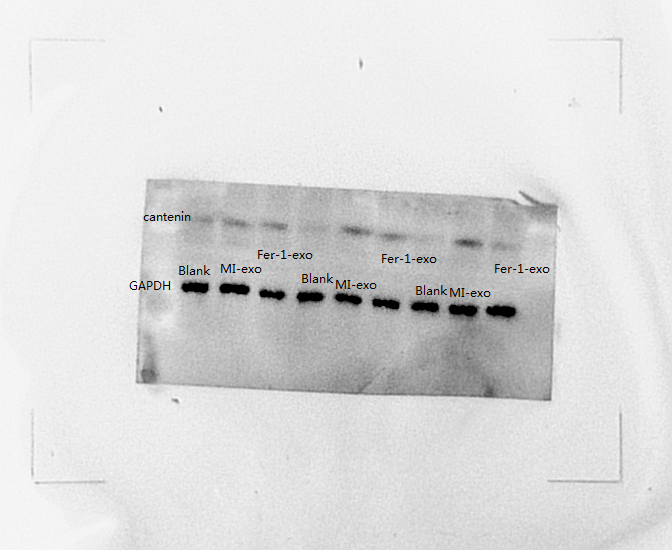

Supplement: Supplemental Information 2 [file peerj-10-13717-s002.zip › 20220316 Raw data/Fig 4D WB gel/GAPDH a┬-catenin.tif]
