# Supplementary material for: Ferroptotic cardiomyocyte-derived exosomes promote cardiac macrophage M1 polarization during myocardial infarction
Source: PeerJ. 2022 Jul 6;10:e13717. doi: 10.7717/peerj.13717 (PMC9270880; doi:10.7717/peerj.13717)
Supplement: Supplemental Information 3 [file peerj-10-13717-s003.docx]

Supplemental Table 1. The primers used in this study.

| Gene | Primers (5’ - 3’) |
| --- | --- |
| WNT1-F | TTCGGCAAGATCGTCAACCG |
| WNT1-R | GCCAAAGAGGCGACCAAAATC |
| U6-F | CGATACAGAGAAGATTAGCATGGC |
| U6-R | AACGCTTCACGAATTTGCGT |
| miRNA-specific stem-loop RT primers: | |
| let-7b-5p-RT | GTCGTATCCAGTGCGTGTCGTGGAGTCGGCAATTGCACTGGATACGACAACCACA |
| let-7c-5p-RT | GTCGTATCCAGTGCGTGTCGTGGAGTCGGCAATTGCACTGGATACGACAACCATA |
| let-7e-5p-RT | GTCGTATCCAGTGCGTGTCGTGGAGTCGGCAATTGCACTGGATACGACAACTATA |
| miR-106b-3p-RT | GTCGTATCCAGTGCGTGTCGTGGAGTCGGCAATTGCACTGGATACGACGCAGCAA |
| miR-129-5p-RT | GTCGTATCCAGTGCGTGTCGTGGAGTCGGCAATTGCACTGGATACGACGCAAGCC |
| miR-146b-3p-RT | GTCGTATCCAGTGCGTGTCGTGGAGTCGGCAATTGCACTGGATACGACCCAGAAC |
| miR-15a-3p-RT | GTCGTATCCAGTGCGTGTCGTGGAGTCGGCAATTGCACTGGATACGACTGAGGCA |
| miR-185-5p-RT | GTCGTATCCAGTGCGTGTCGTGGAGTCGGCAATTGCACTGGATACGACTCAGGAA |
| miR-210-3p-RT | GTCGTATCCAGTGCGTGTCGTGGAGTCGGCAATTGCACTGGATACGACTCAGCCG |
| miR-214-5p-RT | GTCGTATCCAGTGCGTGTCGTGGAGTCGGCAATTGCACTGGATACGACGCACAGC |
| miR-23a-5p-RT | GTCGTATCCAGTGCGTGTCGTGGAGTCGGCAATTGCACTGGATACGACAAATCCC |
| miR-296-5p-RT | GTCGTATCCAGTGCGTGTCGTGGAGTCGGCAATTGCACTGGATACGACACAGGAT |
| miR-324-3p-RT | GTCGTATCCAGTGCGTGTCGTGGAGTCGGCAATTGCACTGGATACGACCCAGCAG |
| miR-381-5p-RT | GTCGTATCCAGTGCGTGTCGTGGAGTCGGCAATTGCACTGGATACGACATATACA |
| miR-433-5p-RT | GTCGTATCCAGTGCGTGTCGTGGAGTCGGCAATTGCACTGGATACGACGAATAAT |
| miR-503-3p-RT | GTCGTATCCAGTGCGTGTCGTGGAGTCGGCAATTGCACTGGATACGACCCTGGCA |
| miR-30c-1-3p-RT | GTCGTATCCAGTGCGTGTCGTGGAGTCGGCAATTGCACTGGATACGACGGAGTAA |
| let-7a-5p-RT | GTCGTATCCAGTGCGTGTCGTGGAGTCGGCAATTGCACTGGATACGACAACTATA |
| let-7d-5p-RT | GTCGTATCCAGTGCAGGGTCCGAGGTATTCGCACTGGATACGACAACTAT |
| miR-150-5p-RT | GTCGTATCCAGTGCGTGTCGTGGAGTCGGCAATTGCACTGGATACGACCACTGGT |
| miR-1911-5p-RT | GTCGTATCCAGTGCGTGTCGTGGAGTCGGCAATTGCACTGGATACGACCCCAACA |
| miRNA primers: | |
| let-7b-5p-F | CAGTGAGGTAGTAGGTTGTGT |
| let-7c-5p-F | GCAGTGAGGTAGTAGGTTGT |
| let-7e-5p-F | GCAGTGAGGTAGGAGGTTG |
| miR-106b-3p-F | CGCACTGTGGGTACTTG |
| miR-129-5p-F | CAGCTTTTTGCGGTCTG |
| miR-146b-3p-F | TGCCCTGTGGACTCAG |
| miR-15a-3p-F | GCAGCAGGCCATATTGTG |
| miR-185-5p-F | CAGTGGAGAGAAAGGCAGT |
| miR-210-3p-F | GCTGTGCGTGTGACA |
| miR-214-5p-F | TGCCTGTCTACACTTGCT |
| miR-23a-5p-F | GGGGTTCCTGGGGATG |
| miR-296-5p-F | GGCCCCCCCTCAATC |
| miR-324-3p-F | CTGCCCCAGGTGCT |
| miR-381-5p-F | GCGAGGTTGCCCTTTG |
| miR-433-5p-F | AGTACGGTGAGCCTGTC |
| miR-503-3p-F | CGCAGGGGGTATTGTTTC |
| let-7a-5p-F | GCGGGGTGAGGTAGTAGGTTG |
| let-7d-5p-F | CGCAGAGAGGTAGTAGGTTG |
| miR-150-5p-F | CTCCCAACCCTTGTACCA |
| miR-1911-5p-F | GTGAGTACCGCCATGTCT |

Note: F means forward primers, R means reverse primers, RT means reverse transcription
